# High Intensity Functional Training (HIFT) Improves Fitness in Recruit Firefighters

**DOI:** 10.3390/ijerph182413400

**Published:** 2021-12-20

**Authors:** Annmarie Chizewski, Allyson Box, Richard M. Kesler, Steven J. Petruzzello

**Affiliations:** 1 Department of Sport and Exercise, College of Education, Benedictine University, Lisle, IL 60532, USA; 2 Department of Kinesiology and Community Health, College of Applied Health Sciences, University of Illinois Urbana-Champaign, Champaign, IL 61801, USA; agbox2@illinois.edu (A.B.); rkesler2@illinois.edu (R.M.K.); petruzze@illinois.edu (S.J.P.); 3 Illinois Fire Service Institute, University of Illinois Urbana-Champaign, Champaign, IL 61820, USA

**Keywords:** firefighter, high-intensity training, fitness, functional training

## Abstract

Background: Firefighting is a strenuous profession requiring adequate levels of fitness for effective job performance. Providing firefighters with a safe and effective fitness program is essential for optimal performance. The purpose of this project was to examine changes in various parameters of physical fitness and firefighter ability following a 7-week high intensity functional training (HIFT) program. Methods: Participants were male firefighter recruits (*N* = 89; age = 27.1 ± 4.2 years, height = 1.78 ± 0.1 m, BMI = 28.1 ± 4.2) enrolled in a Basic Operations Firefighter Academy. Fitness and firefighting ability (via the Academy Firefighter Challenge) were assessed at Weeks 1 and 7 of the Academy. Results: Significant improvements in both fitness and firefighter ability were seen following the HIFT program. Specifically, fitness (BMI, cardiovascular fitness, muscular endurance) improved significantly [Hotelling’s *T*^2^ = 8.98, *F*(5, 84) = 150.92, *p* < 0.001, *η*^2^*_p_* = 0.90]. Firefighter ability also improved significantly [Hotelling’s *T*^2^ = 3.95, *F*(7, 88) = 46.26, *p* < 0.001, *η*^2^*_p_* = 0.80]. Conclusions: Following a 7-week Basic Operations Firefighter Academy that included daily HIFT, significant increases in fitness and firefighting ability were observed. These findings suggest that HIFT appears to be an effective means of improving fitness and firefighting ability in recruit firefighters.

## 1. Introduction

The National Fire Protection Association (NFPA) has recommended that all municipal departments conduct annual physical fitness testing of their firefighter employees and provide them with a physical activity or wellness initiative [1,2]. Despite these recommendations, nearly 70% of municipal departments across the United States do not formally require their employees to maintain physical fitness standards nor do they provide them with an exercise program [3]. Most current full-time firefighters do not meet the recommended level of aerobic capacity (VO_2max_ = 42 ml∙kg^−1^∙min^−1^) to perform job-related duties safely and efficiently [4,5,6]. This profession requires firefighters to be able to complete tasks that require cardiovascular endurance, muscular strength and endurance, power, and agility on a regular basis [7,8,9,10,11]. When firefighters do not possess adequate levels of physical fitness, they jeopardize their own lives, the lives of their shift mates, and the community they serve.

Approximately 50% of firefighters are classified as overweight with an additional 25% being classified as obese [12,13,14]. Overweight and obese firefighters are at higher risk of experiencing injury, missing work, suffering from chronic illness, and experiencing a cardiac incident compared to their healthy weight peers [9,13,15,16]. Combined with the lack of physical fitness, overweight and obese firefighters create an even greater level of concern. In addition to physical health issues, mental health issues are prevalent in the fire service. Given that exercise has been shown to be an effective way of treating these health issues in the general population, physical activity and exercise may improve the mental health in first responders as well. By providing firefighters with fitness initiatives and physical activity programs, municipal departments could reduce the risk of cardiovascular incidents and chronic diseases (i.e., hypertension, diabetes, anxiety, depression, etc.) [5,17,18,19,20,21].

Additionally, research has begun to explore the relationship between physical fitness and performance on firefighting-specific tasks. These findings suggest that individuals who possess higher levels of physical fitness can perform simulated firefighting drills more efficiently when compared to their less fit peers [21,22,23,24]. Firefighters who possessed higher levels of physical fitness performed better on simulated fireground tasks, indicating a relationship between fitness level and ability to firefight. It is imperative to provide firefighters with functional physical training programs that are designed to not only improve physical fitness, but that also improve job performance.

High intensity training has been gaining popularity among the tactical athlete population (e.g., firefighters, law enforcement officers) in recent years [25]. One form of high intensity training that could prove beneficial to firefighters is High Intensity Functional Training (HIFT). HIFT can be defined as a training style that utilizes a variety of training modalities which are performed at a high-intensity and are designed to improve the various parameters of general physical fitness [26,27,28,29]. HIFT varies from traditional exercise programming and high-intensity interval training which are unimodal and often only focus on one aspect of fitness in a workout. HIFT targets multiple facets of fitness in a single workout (i.e., strength, aerobic capacity, power, etc.). Functional movements can be defined as movements that involve the whole body, universal motor recruitment patterns, and exercises performed in different planes of movement (i.e., frontal, sagittal, and transverse) [27,28,29]. Universal motor recruitment patterns can also be defined as patterns that are used in everyday life and needed for normal function. Simply put, universal motor recruitment patterns involve the use of all muscles moving in symphony efficiently, effectively, and quickly to complete a given task. This involves everything from large main muscles used in a specific movement to all the small supporting muscles that stabilize the movement.

For firefighters specifically, their work requires them to possess multiple aspects of fitness, as well the ability to use both type I (slow twitch) and type II (fast twitch) muscle fibers. Movements such as forcing entry, raising ladders, and advancing hose lines require short powerful bouts of work (~10 s) and require the phosphagen energy system. Tasks such as climbing stairs, searching, performing overhaul, and extended hose operation require longer bouts of work and utilize oxidative energy systems (>120 s; [30,31]). Firefighters face physical challenges on a regular basis and should possess an adequate and well-rounded battery of physical fitness attributes (e.g., cardiovascular endurance, muscular strength, muscular endurance, flexibility). Given its constantly varied and dynamic nature HIFT may be an effective training modality for developing or maintaining such attributes. HIFT programs generally require full-body movement and multi-joint coordination which would not only contribute to increases in fitness but also the movement literacy firefighters. Improving movement literacy and quality may reduce the number of injuries experienced as well.

Previous work has highlighted the effectiveness of HIFT in military populations. In boot camp, soldiers completing a HIFT training program had an increased number of push-ups, bench-press repetitions, and increased flexibility, as well decreased 2-mile run time and step test heart rate compared to soldiers completing the more traditional Army Physical Readiness Training (APRT) program [29]). HIFT has been shown to be effective in improving fitness in several military samples [28,29]. Given that there are abundant evidence-based benefits for HIFT programs in military personnel, there are no pressing reasons why the numerous benefits would not be applicable to other tactical athletes such are firefighters. Significant reductions in injuries in military personnel have been shown when comparing HIFT to traditional physical training [29]. HIFT may lead to reductions in injuries, increased fitness levels, and may reduce the number of line-of-duty deaths due to cardiac incidents and overexertion. Little to no research has been conducted examining HIFT within a firefighter population. However, given that there is a link between fitness and job performance in firefighters, using HIFT to increase fitness may also lead to increases in firefighting ability.

High intensity exercise methods are rapidly gaining popularity across the globe and across numerous populations [27,28]. HIFT programs, specifically, target multiple fitness domains and often yield improvements in physical and mental readiness. As a result of this, HIFT seems to be an appropriate training modality among tactical athletes who face a constantly changing environment [28,29]. Tactical athletes such as military personnel and first responders are required to possess at least adequate levels of strength, speed, power, endurance, and agility. Due to the demands tactical athletes face, being able to prescribe an appropriate and effective fitness program is essential for the health and safety of these men and women. While some research has compared the effectiveness of HIFT versus more traditional unimodal training in military populations [29], little to no research has been conducted to examine the impact HIFT has on fitness and job performance in firefighters.

The present study is one of the first to examine the effectiveness of HIFT for increasing firefighting ability, which should translate to better job performance. The purpose of the present study was to examine the changes in physical fitness and firefighting ability in recruit firefighters following a 7-week Basic Operations Firefighter Academy that incorporated daily (60-min, 5 days·wk^−1^) HIFT. It was hypothesized that both fitness (body composition, cardiovascular endurance, muscular endurance, power, and flexibility) and firefighting ability (performance on the Academy Firefighter Challenge) would significantly improve following the 7-week training Academy and the HIFT program that was an integral part of it. Additionally, we sought to compare the effectiveness of the HIFT program compared to the previous, more traditional physical training program. We hypothesized that HIFT training would yield greater improvements in fitness compared to more traditional forms of physical training.

## 2. Materials and Methods

### 2.1. Participants

The present study was approved by the Institutional Review Board at a Midwestern university. The participants were firefighter recruits enrolled in the Basic Operations Firefighter Academy held in the Midwest. Academies are held biannually each year (Fall, Spring); data for this project were collected during the Spring 2018 (*n* = 22), Fall 2018 (*n* = 31), and Spring 2019 (*n* = 36) Academies. The participants were newly hired recruit firefighters (*n* = 89) from the state of Illinois. Due to the low number of female firefighter recruits during this period (*n* = 1), only males were included in the data analysis. See Table 1 for descriptive and fitness data at Week-1. There were no significant differences in participant characteristics (age; *df* = 2, *F* = 0.70, *p* = 0.5, weight; *df* = 2, *F* = 0.44, *p* = 0.65, height; *df* = 2, *F* = 2.38, *p* = 0.10, and BMI; *df* = 2, *F* = 0.33, *p* = 0.98) between the 3 recruit class cohorts. Further, at Week-1 all 3 cohorts had an average estimated VO_2max_ that was classified as “poor” according to the American College of Sports Medicine Cardiorespiratory Fitness Classifications [32].

### 2.2. Daily HIFT

As part of the 7-week Academy program, participants engaged in a daily HIFT program (60 min∙d^−1^; 5 d∙wk^−1^) designed to improve both fitness and firefighting ability. HIFT took place on the Academy training grounds. The functional fitness program was developed and supervised jointly by the research staff at the Fire Academy, exercise specialists from the Exercise Psychophysiology Laboratory (ExPPL) of the local university, and from consultations with a certified CrossFit^®^ coach. The program supervisors provided coaching on technique and form while also providing encouragement and directions. The program incorporated high intensity bouts of work where the recruits were asked to work as hard as they could in a given timeframe or complete a specific task as quickly and safely as possible. High intensity was defined as working at a heavy to severe intensity (at or above lactate threshold). Heavy intensity exercises were sustained for less than 30 min, while severe intensity exercises lasted for less than 10 min at a time. These short, intense bouts of work were typically followed with a rest period while their partner or a different company completed the assigned task.

Firefighter recruits reported to the Academy campus at 0630 every weekday morning of the 7-week Academy for physical training (PT). Daily PT began with roll call and a dynamic warm-up (which took approximately 10 min). This included jumping jacks, jump rope, and dynamic stretching. Following the warmup, the recruits were led through approximately 40-min of HIFT. The activities varied daily, and highlighted aspects of fitness required for safe and efficient firefighting: aerobic capacity, muscular strength and endurance, power, flexibility, and agility. The program gradually incorporated movements and equipment commonly used during firefighting tasks (e.g., hoses, sledgehammer) and is detailed in Table 2 below.

We also examined whether any group differences occurred between the current HIFT program and the previous, traditional physical training (PT) program. This traditional PT program emphasized cardiovascular training (See Table 3 for previous program details). From a sample of 500+ individuals who were previously enrolled in the Basic Operations Firefighter Academy and had participated in the PT program, we approximated age and estimated VO_2max_-matched controls for everyone who participated in the HIFT program during the current study. If multiple matched controls were found per participant, they were then best matched by BMI. The previous PT program also occurred at the beginning of the day and only occurred 4 days per week for 30 min. The PT began with a 10-min warm-up phase which included static stretching and callisthenic exercises. Following the warm-up, the participants took part in a 20-min conditioning phase which focused primarily on cardiorespiratory endurance but also included total body conditioning. Daily PT was concluded with a 5-min cool down phase which focused on stretching and returning heart rates to resting state.

The outcome measures for the HIFT and traditional PT programs were taken over the course of 3 days during Week-1 and Week-7. These included: (a) age, height, weight; (b) fitness (1.5-mile (2.4 km) run time, number of push-ups completed in 60-s, number of sit-ups completed in 60-s, number of repetitions completed during the Young Men’s Christian Association (YMCA) Bench Press test, sit and reach, and vertical jump height); and (c) Firefighter ability (time to complete the Academy Firefighter Challenge, AFC). Baseline testing took place on 3 days during Week 1 and post testing took place during Week-6 and Week-7 of Academy. Day 1 of testing consisted of the 1.5-mile (2.4 km) run, 60-s push-ups, and 60-s sit-ups; Day 2 consisted of measures of height, weight, vertical jump, YMCA bench press, and sit and reach; and Day 3 consisted of the AFC. Mid-point measures of cardiovascular fitness (1.5-mile (2.4 km) run) and muscular endurance (60-s push-ups, 60-s sit-ups) were also obtained at Week 4.

### 2.3. Physical Fitness Testing

Measurements of health and fitness were collected at Week-1 and Week-7. The data collection was carried out by the ExPPL research staff and was supervised by Academy instructors. Rest time was given between physical fitness assessments on Day 1 and Day 2 of testing to reduce the likelihood of muscular fatigue. The same researchers conducted the testing throughout the academy to ensure inter-rater reliability. Additionally, all test administrators had to undergo detailed training that was supervised by ExPPL research leaders to ensure tests were being administered and monitored appropriately.

Day 1. Muscular endurance and aerobic capacity were tested on Day 1. The first test administered was the 60 s push-up test. The recruits were instructed to complete as many push-ups as possible in 60 s. Another recruit kept track of the correct number of repetitions completed and helped record the score. The recruits were required to bend their elbows and lower their entire body as a single unit until their upper arms were at least parallel to the ground. The recruits then fully extended their arms returning their body as a single unit to the starting position. The only permitted resting position was upward into a pike. The test was immediately stopped if any portion of their body (e.g., stomach and knee) touched the floor, and only repetitions completed prior to that occurring were reported. This format of testing was the standard used in previous academies and was adopted for testing purposes during data collection.

The recruits then were asked to complete as many sit-ups as possible in 60 s (following a period of rest between). The recruits assumed the starting position by lying on their back with their knees bent at a 90-degree angle. They could position their feet together or up to 12 inches apart while another person held their ankles with only their hands. No other method of bracing or holding the feet was authorized. The recruits were required to keep their fingers interlocked behind their heads and the heel was the only part of their foot that had to stay in contact with the ground. When the recruits were given the command “Go”, they began to raise their upper body forward to, or beyond, the vertical position (i.e., the base of their neck was above the base of their spine). After they reached or surpassed the vertical position, they lowered their upper body until the bottom of their shoulder blades touched the ground. Their head, hands, arms, or elbows did not have to touch the ground. Repetitions were not counted if they failed to keep their fingers interlocked behind their head, arched or bowed their back, raised their buttocks off the ground, let their knees exceed a 90-degree angle, or failed to reach the vertical position. This format of testing was the standard used in previous academies and was adopted for testing.

For assessment of aerobic capacity (ml∙kg^−1^∙min^−1^), the recruits completed a 1.5-mile (2.4 km) run on a pre- determined course. They were instructed to complete the course as quickly as they could, but the pace was self-selected. Estimated aerobic capacity was determined using the formula: (3.5 + 483/1.5-mile time (min)) [32].

Day 2. Weight (i.e., body mass) and height (without shoes) were recorded in kilograms and centimeters, respectively, on a Seca 284 digital scale. Body Mass Index was calculated using the formula (kg∙m^−2^; [32]).

The Golding et al. [33] YMCA bench press protocol was used to assess muscular endurance. The participants were told to complete as many repetitions as possible, raising and lowering the barbell to the beat of a metronome set to 60 b∙min^−1^ or until they reached a maximum of 60 repetitions. The total weight of the barbell was 80 lbs (36 kg).

Hamstring and lower back (trunk) flexibility was assessed using a sit and reach box [32]. The participants sat on the floor with legs stretched out, backs of the knees flat on the floor, and soles of the feet (without shoes) flat against the back of the sit-and-reach box. With hands placed one on top of the other, they were instructed to reach as far forward as possible in one smooth motion. Participants were given three tries and the best of three was recorded for their final score.

Vertical jump was assessed as a measure of power [34]. Each recruit stood near the Jump USA Vertec Vertical Jump System with one arm fully extended so the tester could record their standing height. They then jumped up and touched the highest possible vane of the Vertec System. The jump height was the difference between standing height and jumping height. Participants were given three tries and the best of three was recorded for their final score.

Day 3. Firefighter ability was assessed at Weeks 1 and 7 via the Academy Firefighter Challenge (AFC). The AFC is a six-event physical performance test used to assess cardio-respiratory fitness and muscular endurance. The firefighters wore full turnout gear, which included helmet, bunker pants, bunker coat, boots, gloves, and Self-Contained Breathing Apparatus (SCBA) equipment. The AFC consisted of the following events: Forcible Entry, SCBA Crawl, Victim Drag, Hose Advance, Equipment Carry, and Ladder Raise. An ExPPL research staff member recorded the time to complete each event and the overall time, while also directing the recruit between stations.

Station 1: Forcible Entry. This event required the recruits to use a 9-pound (4.09 kg) sledgehammer to strike the measuring device (Keiser^®^ Sled, Keiser Corporation, Fresno, CA, USA) repeatedly until the sled was moved 5-feet (1.5 m). There was a 90-s time cap for this event.

Station 2: SCBA Crawl. The recruits entered the SCBA course, laid out in a sea land shipping container, and crawled through the darkened course on hands and knees. Besides low visibility, the course was cleared of any obstacles (i.e., hose, confined entryways, etc.); however, the height and width of the maze varied as the recruits progressed through the maze.

Station 3: Victim Drag. The recruits were required to carry or drag a 110-pound (49.90 kg) mannequin 100-ft (30 m) to a pre-marked endpoint. They were allowed to grasp the mannequin in whichever manner they preferred.

Station 4: Hose Advance. This event required the recruits to grasp a 100 ft (30 m), 1¾ inch (44-mm) charged hose and drag the hose 75 feet (22.9 m) to a prepositioned cone. They were allowed to place the hose line over their shoulders or across their chest, but no more than 8-ft (2.4 m) of the hose was permitted to be placed over their body.

Station 5: Equipment Carry. The recruits picked up 2 chainsaws (~15 lbs (6.8 kg) per saw), one in each hand, and carried them 50-ft (15 m) to a designated turn-around point. They were permitted to place the saw(s) on the ground and adjust their grip if needed. Once the firefighters returned to the starting line, they placed each saw on the ground and proceeded to the final station.

Station 6: Ladder Raise. The recruits approached a prepositioned 28 ft (8.5 m) 2-section fixed ladder (i.e., attached at the bottom) that was lying flat on the ground. They were instructed to raise the ladder as quickly as possible from the ground to a fixed vertical position (90 degrees) from its initial position.

### 2.4. Data Analysis

Descriptive statistics were calculated for each of the variables under consideration. In addition, to compare the effects the traditional PT program and the current HIFT program, overall fitness was calculated as a composite score comprised of ΔVO_2max_, ΔYMCA Bench Press, Δ60 s push-ups, Δ60 s sit-ups, and Δflexibility.

To assess changes over the course of 7 weeks, separate repeated measures MANOVAs were conducted: (a) one included body composition, muscular endurance variables (i.e., YMCA bench press, 60-s push-ups, 60-s sit-ups), and cardiovascular endurance (1.5-mile run time); and (b) one for firefighting ability (i.e., Keiser sled, SCBA crawl, victim drag, hose advance, equipment carry, ladder raise, and total completion time). Variables that had a moderate correlation (0.3–0.7) were grouped together for the MANOVAs to reduce Type I error [35]. Variables with a correlation <0.3 were unrelated and anything >0.7 was interpreted as indicative of redundancy. Following a significant MANOVA, univariate tests were conducted to determine which of the dependent measures changed significantly and to what extent. Univariate repeated measures ANOVAs were run for flexibility (sit and reach) and vertical jump due to the uniqueness of each variable. A final MANOVA was run to determine the time effect from Week 1 to Week 4, and Week 4 to Week 7 for the following measures: cardiovascular endurance, push- ups, and sit-ups.

A significance level of *p* < 0.05 was chosen to denote statistical significance. Partial *η*^2^ (*η*^2^*_p_*) was calculated as a measure of effect size (0.02 = small effect; 0.13 = medium effect; 0.26 = large effect). Cohen’s d (*d*) was calculated as measure of effect size (0.20 = small effect size; 0.50 = medium effect size; 0.80 = large effect size) for these variables. Statistical analyses were conducted using SPSS 24.0 for Windows (SPSS, Chicago, IL, USA).

## 3. Results

A repeated measures MANOVA including body composition (BMI), cardiovascular endurance and muscular endurance (# of repetitions of push-ups, sit-ups, and YMCA bench press) revealed significant improvements from Week 1 to Week 7 [Hotelling’s T^2^ = 8.98, *F*(5, 84) = 150.92, *p* < 0.001, *η* = 0.90; see Table 4]. Univariate tests for each dependent measure revealed that each changed significantly from Week 1 to Week 7 (BMI, [*F*(1, 88) = 4.6, *p* < 0.05, *d* = 0.04] 1.5-mile (2.4 km) run time: [*F*(1, 88) = 132.66, *p* < 0.001, *d* = 0.85]; push-ups: [*F*(1, 88) = 369.47, *p* < 0.001, *d* = −1.22]; sit-ups: [*F*(1, 88) = 143.44, *p* < 0.001, *d* = −0.99]; bench press: [*F*(1, 88) = 82.04, *p* < 0.001, *d* = −0.45]). Univariate repeated measures ANOVAs were run for flexibility (sit and reach) and lower body power (vertical jump). Time effects for flexibility revealed significant changes from Week 1 to Week 7 [*F*(1, 88) = 51.07, *p* < 0.001, *d* = −0.33]. Time effects for lower body power were not significant (*p* > 0.05).

A MANOVA revealed significant (*p* ≤ 0.001) time effects from Week 1 to Week 4 and Week 4 to Week 7 for cardiovascular endurance (1.5-mile (2.4 km) run) and muscular endurance (60 s push-ups and 60 s sit-ups) [Hotelling’s T^2^ = 7.92, *F*(6, 76) = 100.27, *p* < 0.001, *η*^2^*_p_* = 0.89]. Univariate tests revealed significant (*p* < 0.001) time effects for cardiovascular endurance (*η*^2^*_p_* = 0.52), push-ups (*η*^2^*_p_* = 0.69), and sit-ups (*η*^2^*_p_* = 0.49). Cardiovascular endurance significantly changed from Week 1 to Week 4 [*p* < 0.001, d = 0.46] and again from Week 4 to Week 7 [*p* < 0.001, *d* = 0.38]. Time effects for push-ups were also significant from Week 1 to Week 4 [*p* < 0.001, *d* = −0.66] and from Week 4 to Week 7 [*p* < 0.001, *d* = −0.52]. Finally, a significant time effect for sit-ups was shown from Week 1 to Week 4 [*p* = 0.001, d = −0.30] and from Week 4 to Week 7 [*p* < 0.001, *d* = −0.62].

Additionally, at baseline, 37% of the present sample was classified as having “poor” or “very poor” aerobic fitness based on ACSM Fitness Category determined from their estimated VO_2max_ and age [32]. Roughly 50% of the sample was classified as “fair” and less than 10% possessed “good” aerobic fitness. Following the 7-week HIFT program, nearly 35% of the sample was classified as having “excellent” or “good” aerobic fitness. Additionally, the number of individuals who were classified as “poor” or “very poor” was reduced to 11% following the 7-week intervention (See Table 5 for Specifics).

A final repeated measures MANOVA was run to examine the change in both firefighting ability (time for each of Keiser^®^ sled, SCBA crawl, victim drag, hose advance, equipment carry, ladder raise, and total completion time) from Week 1 to Week 7 [Hotelling’s T^2^ = 3.95, *F*(7, 82) = 46.26, *p* < 0.001, *η*^2^*_p_* = 0.82]. Univariate tests for each dependent measure revealed significant improvements in completion time (i.e., faster) for the Keiser^®^ sled [*F*(1, 88) = 33.62, *p* < 0.001, *d* = 0.62], SCBA crawl [*F*(1, 88) = 81.43, *p* < 0.001, *d* = 0.88], victim drag [*F*(1, 88) = 44.05, *p* < 0.001, *d* = 0.59], hose advance [*F*(1, 88) = 5.94, *p* < 0.05, *d* = 0.35], equipment carry [*F*(1, 88) = 35.06, *p* < 0.001, *d* _=_ 0.51], ladder raise [*F*(1, 88) = 16.66, *p* < 0.001, *d* _=_ 0.48], and total completion time [*F*(1, 88) = 207.16, *p* < 0.001, = 1.16] from Week 1 to Week 7.

The traditional PT training program and HIFT training program both resulted in significant improvements in overall fitness. There were no significant differences in baseline fitness measures when comparing the HIFT program to the PT program previously used. The HIFT program resulted in significantly greater changes in 1.5-mile (2.4 km) run time, estimated VO_2max_, 60 s sit-ups, YMCA bench press, and overall fitness, when compared to the past program (see Table 6 for statistics). The HIFT program resulted in a greater Δ60 s push-ups with a larger effect size when compared to the PT program; however, the differences were not statistically significant.

## 4. Discussion

The present study had 3 primary goals: (a) examine changes in multiple aspects of physical fitness following a 7-week HIFT program; (b) examine changes in Firefighter ability following a 7-week HIFT program; and (c) compare the effectiveness of the current HIFT training program with the previous, traditional physical training program. Results revealed significant improvements in weight (i.e., body mass) and BMI as well as various aspects of physical fitness (cardiovascular endurance, muscular endurance, and flexibility). Additionally, significant improvements in Firefighter ability (Keiser^®^ sled, SCBA crawl, victim drag, hose advance, equipment carry, and total completion time on the AFC) were observed following the 7-week Basic Operations Firefighter Academy that included the HIFT program. Lastly, the HIFT program was more effective in improving fitness than the cardio-endurance heavy traditional PT program. These findings highlight and support previous research which showed that HIFT can be an effective training style to improve both fitness and job performance [17,26,28,29,30,31].

Firefighting is a profession that requires a high level of physical fitness for optimal performance on the fire-ground. Most career firefighters do not meet the required aerobic capacity or physical fitness needed to perform work-related duties most effectively [5,11,21,23,36]. Previous work has suggested that a VO_2max_ of >42 ml∙kg^−1^∙min^−1^ is needed to perform firefighting tasks safely and effectively [6]. At Week 1 of the present study, nearly 55% of the participants did not possess the suggested minimum level of aerobic fitness, with the average estimated VO_2max_ being 40.8 ml∙kg^−1^∙min^−1^. By Week 7, the average estimated VO_2max_ was 45.3 ml∙kg^−1^∙min^−1^. The number of recruits not having the suggested minimal level of aerobic fitness was reduced by >50%, with only 26% not meeting the recommended VO_2max_. From Week 1 to Week 7, the number of participants who were classified as “excellent”, “good”, and “fair” for aerobic fitness increased and the number of individuals classified as “poor” and “very poor” decreased (Table 4). These results highlight the effectiveness of HIFT training and emphasize that constantly varied functional training can increase aerobic capacity, along with other components of fitness. Our findings support previous work showing HIFT results in significant improvements in aerobic capacity [28,29,30]. In a review by Haddock et al. [29] of multiple studies, results revealed significant improvements in aerobic fitness after a HIFT intervention ranging from 8–12 weeks.

Similarly to the present study, Cornell et al. [37] had 27 male firefighter recruits (29.9 ± 4.1 years; 179.8 ± 4.6cm; 87.2 ± 9.7 kg) enrolled in a 14-week training academy, part of which included a physical training component. They showed significant changes from Week 1 to Week 14, with decreases in body mass (3%), waist-to-hip ratio (2%), and estimated body fat (31%). There was also a 4% increase in fat-free mass, a 28% increase in relative VO_2max_, increases in muscular strength (9% for bench press, 32% for squat), and increased muscular endurance (36% increase in maximum push-ups). Cornell et al. utilized a more traditional physical training program that focused on aerobic capacity, with ability group runs taking place 2–3 times per week. On weeks when the recruits only ran 2-times, they completed a 30-min stair climb run. The training program also incorporated traditional weight training, which followed a linear progression of three sets of 8 to 10 repetitions at 60–80% of the individual’s estimated one-repetition maximum (1-RM) 2–3 times per week [37]. Our programming utilized HIFT during a 7-week training Academy and saw similar fitness improvements. We observed an 11.0% increase in aerobic capacity along with significant increases in muscular endurance (specifically, a 37.2% increase in push-ups, a 22.0% increase in sit-ups, and a 17.1% increase in number of YMCA bench press repetitions). Additionally, we observed a 28.9% increase in flexibility. The HIFT program in the present study incorporated equipment and movements that are often used in the fire service. Significant improvements in cardiovascular and muscular endurance were seen from Week 1 to Week 4, with small to moderate increases based on effect sizes (*d*_s_ = 0.34–0.44). These results suggest that fitness may improve in as little as 4 weeks using HIFT. Significant improvements continued from Week 4 to Week 7 in our HIFT programming. Given that these findings suggest that improvements in fitness can occur in as little as 4 weeks using HIFT programming, it is plausible that HIFT is just as, if not more, effective when compared to traditional forms of exercise which focus on one training modality.

HIFT has shown to be an effective means to improve fitness in military personnel. Heinrich et al. [29] utilized a HIFT program and compared its effectiveness to the standard Army Physical Readiness Training (APRT) program. Thirty-four active-duty personnel were assigned to a HIFT program and 33 were assigned to the APRT program. The training programs occurred 2 times per week over the course of 8 weeks. The participants who engaged in HIFT significantly improved their push-ups by an average of 4.2 (±5.4) compared to 1.3 (±5.9) push- ups for the APRT group [29]. Additionally, the HIFT group saw significantly greater improvements in their 2-mile (3.2 km) run times, bench press strength, and flexibility when compared to the APRT group. The HIFT group experienced significant decreases in heart rate (−17.0 ± 15.0 b·min^−1^ vs −9.0 ± 16.1 b·min^−1^) on the step test compared to the APRT group. We found similar increases in fitness in firefighters using HIFT over the course of 7-weeks during a Basic Operations Firefighter Training Academy. Taken together these findings suggest that HIFT may be an effective form of training for tactical athletes such as firefighters.

Roberts et al. [10] utilized a 16-week traditional aerobic training program to increase aerobic capacity in a large sample of firefighters (*n* = 115). The results revealed that prior to the initiation of the fitness program, aerobic capacity (average = 35 ± 7 ml∙kg^−1^∙min^−1^) was 20% lower than what is deemed sufficient for safe firefighting. Roberts et al. [10] found that following the 16-week program, aerobic capacity was increased by 28% to an average of 45 ± 6 ml∙kg^−1^∙min^−1^. The firefighters in our study started at an average baseline estimated VO_2max_ of 40.8 ± 5.1 and after 7-weeks of HIFT it was increased to 45.3 ± 5.2 ml∙kg^−1^∙min^−1^. Our cohort had higher baseline fitness levels but was still able to significantly increase their aerobic capacity to similar levels in just 7 weeks of HIFT compared to 16 weeks of aerobic training. Additionally, the HIFT program increased muscular endurance and flexibility by 37.2% and 28.9% compared to 19.6% and 2.9%, respectively, in the 16-week intervention. Significant changes in both cardiovascular (*d* = 0.46) and muscular endurance (push-ups: *d* = −0.66; sit-ups: *d* = −0.30) were observed in as little as 4 weeks using HIFT in our study. Our findings suggest that HIFT may potentially be a more effective means of improving various components of fitness among firefighters than traditional aerobic training programs used in the past.

We also hypothesized that firefighter ability would significantly improve following the 7-week HIFT program. Very little previous research has examined the impact of a fitness intervention on firefighter ability. However, similar findings have been observed in military personnel. Our findings add to the growing body of evidence that suggests improvements in physically demanding job performance can be observed following a non-traditional physical fitness training program [21,38]. Besides increased fitness, our results also revealed significant improvements in firefighter ability (i.e., firefighter-specific tasks routinely performed on the fire- ground). Specifically, we observed improvements in performance on the Keiser^®^ Sled (−20.1%; negative percentage indicated faster Week 7 times compared to Week 1), SCBA Crawl (−20.4%), victim drag (−13.8%), hose advance (−8.6%), equipment carry (−7.7%), ladder raise (−12.2%) and total AFC completion time (−19.9%). These findings are noteworthy because of the physical demands that accompany firefighting. When firefighters fail to maintain adequate fitness levels, they jeopardize not only their own well-being, but also their co-workers and the victims they are responsible for [11,22,39,40]. Improving physical fitness has the potential to improve worksite productivity, efficiency, and safety of firefighters by increasing their ability and reducing the risk of experiencing injury while on duty. However, because the firefighters were participating in the Basic Operations Firefighter Academy as well as the HIFT program, it is challenging to determine whether the increases in firefighter ability were due to the HIFT, the training academy, or most likely a combination of both.

Previous work has examined improvements in firefighter job performance following a fitness intervention. A total of 14 well-trained firefighter academy attendees (21.9 ± 1.8 years; 180.9 ± 5.7 cm; 85.6 ± 9.9 kg) took part in a 9-week physical fitness intervention [38]. During the 9-week training program (60–90 min; 3 times·wk^−1^), the firefighters were separated into either undulation training (UT) or standard training groups. The UT model utilized daily fluctuations in training to elicit one or more components of fitness (i.e., muscular endurance/hypertrophy, muscular strength, power output, etc.). The standard training group utilized a more traditional periodization model [39]. Measures of physical fitness and firefighter ability (Grinder performance) were taken at baseline and post-intervention. The UT followed a 3-day upper and lower body program that varied the focus (i.e., endurance/hypertrophy, strength, or power and speed) of exercises each day and the standard training incorporated three 3-week mesocycles that each focused on different aspects of fitness (i.e., endurance/hypertrophy, strength, power and speed) [38]. The Grinder test measured firefighter ability via six tasks that included an equipment hoist, a hose-pull, a Keiser^®^ Sled, a stair-climb, an attic crawl, and a simulated civilian carry or drag. Both training groups experienced significant improvements in fitness from baseline to post-intervention, including 1-RM bench press, 1-RM squat, power output, and Grinder performance time. The individuals who participated in the UT experienced significantly greater improvements in Grinder performance when compared to the traditional training group. Generally, UT elicited greater improvements on the administered test, including upper-body muscular strength, and lower-body muscular strength [38]. These results suggest that a more varied exercise program may be more effective in improving fitness and job performance compared to a more standard (i.e., traditional) training style. Our study utilized a similar all-male sample of recruit firefighters and found similar improvements (~20%) in firefighter ability following a varied functional training program.

Given the extreme demands placed on firefighters, being able to prescribe exercise safely and effectively is vital for the safety and well-being of these individuals. HIFT programs aim to target multiple fitness domains, which could potentially lead to an improvement in the physical readiness in the individuals who utilize this form of training [27,28,29]. HIFT seems to be an appropriate training strategy for firefighters who are constantly faced with a changing environment that requires physical readiness. Our study highlights the positive impact a non-traditional fitness program may have on firefighter fitness and job performance. The HIFT program utilized in our study shared similar results to previous research that examined the effectiveness of HIFT compared to traditional training programs among military populations. [28,29]. They found that HIFT was as effective if not more effective than traditional unimodal training programs previously used. Perhaps providing firefighters with a more functional program such as HIFT could increase enjoyment, engagement, and increase adherence when compared to more traditional styles of training. Currently, the NFPA has recommended that all municipal departments conduct annual physical fitness testing and provide their firefighter employees with a physical activity or wellness initiative [1,2], yet only 30% of municipal departments across the United States formally require their employees to maintain physical fitness standards or provide them with an exercise program [3]. What is currently being prescribed or recommended among firefighters is not working, given that many current full-time firefighters do not meet the recommended fitness standards [4,5,6].

The main limitation of the present study was the lack of a control or comparison group. This limitation is two-fold. First, it is impossible to isolate the effect of the HIFT training on fitness and firefighting ability improvements as recruits were simultaneously engaged in Basic Operations Firefighting Academy. As a result of this, it is unknown whether the gains and improvements in fitness and firefighter ability were due to the HIFT, the Firefighter training academy [which included manual labor and fire-ground training (live fires, equipment hoist and carry, ladder work, etc.)], or a combination of both. However, despite participating in 8+ hours of physically demanding fire-ground training 5 days·wk^−1^ for 7 weeks and self-reported fatigue and muscle soreness, significant improvements were seen in various components of fitness. Second, by necessity all participants needed to complete the same physical training each morning, forcing us to compare fitness changes from the current HIFT cohort to previous traditional PT cohorts.

Future studies could observe the changes in firefighter ability following a 7-week HIFT program compared to a control group. Researchers could utilize a no-exercise control group which would not participate in HIFT but would participate in the Basic Operations Firefighter Academy. Alternatively, changes in fitness and firefighter ability can be observed in current career firefighters who receive a 7-week HIFT program. Additionally, future research could utilize a cross-over design, where half the group engages in daily HIFT during the Academy for 3.5 weeks, and the other group receives a lecture-based curriculum emphasizing the important of physical fitness and health for 3.5 weeks. The groups would then switch halfway through to see whether any observed changes are due to the HIFT or from training they are receiving on the fire-ground. Our study utilized one HIFT prescription for all of the participants in the study. Future research could also include more individualized programs based on the participant’s baseline fitness testing. Providing more individualized programs may yield even more positive changes in fitness and could also lead to greater levels of enjoyment and program adherence.

An additional limitation of the study was that exercise intensity was not systematically measured during the HIFT training. While recruits were instructed to work as hard and quickly as possible, we cannot be certain that the intensity at which the activities were performed was high intensity (>70% of estimated maximal heart rate). Future studies should utilize heart rate monitors to ensure the intensity could be classified as high intensity. Another possible solution, that might be more cost effective than monitoring heart rate, is the use of self-reported rating of perceived exertion (RPE). High intensity interval training has been shown to be as, or more, effective in increasing overall fitness than traditional training programs, as well as being perceived as more enjoyable despite working at relatively high exercise intensities [27,28,29,30]. Future research is warranted to further explore the positive benefits HIFT might have on both fitness and firefighter.

We did not have a measure of muscular strength due to the time constraints that were in place for testing. Testing needed to be completed in 60 min and we had 25–40 recruits to test during this time frame. We were unable to conduct gold standard muscular strength testing (i.e., 1-RM bench press, and/or 1-RM back squat). However, research has shown the YMCA bench press test to be a reliable test that can be used to predict 1-RM bench press for men and women [41]. Given that many of the tasks performed on the fire-ground (i.e., equipment carry, ladder raise, hose hoist, etc.) require muscular strength to perform optimally, future research could incorporate a gold standard measure of muscular strength in order to observe whether changes occur in muscular strength following a HIFT program.

Another potential limitation of the present study and current literature is the lack of female participants. Given that males make up over 95% of those in the fire service, it is challenging to find a large enough sample of female firefighters to observe and include in such research. An additional limitation of the present study is that participants were required to engage in structured, daily physical activity for 7 weeks (60-min, 5 days∙week^−1^) as a condition of graduating the Academy. As such, dropout rates were low and not an accurate depiction of what occurs once firefighters graduate the Academy and are active-duty fire service employees. Previous research has shown that adherence rates for active-duty firefighters are low and improvements that occur during a training academy are largely lost after 6 months on duty [37]. Given this information, future research should also target career firefighters who are currently not required to engage in regular physical activity or fitness programming.

Lastly, we did not examine injuries, movement literacy, or movement quality. HIFT is likely to improve movement literacy due to the full-body movements and multi-joint coordination that it offers and that is required on the fireground. Future research should also examine the movement quality/patterns before and after a HIFT program to determine if movement quality is improved in addition to physical fitness. By improving movement quality and literacy, HIFT may also prove beneficial in reducing on-duty injuries that are commonly seen among firefighters. Increasing fitness and reducing the number of injuries on the fireground would ultimately lead to healthier firefighters which could lead to safer communities.

## 5. Conclusions

The present study provides evidence that HIFT training can be a safe and effective means of improving fitness and firefighter ability in recruit male firefighters. Given that movements seen on the fireground require multiple aspects of fitness, firefighters should be encouraged to engage in physical fitness programs that incorporate all aspects of fitness. HIFT utilizes an assortment of training modalities performed at a high-intensity and are designed to improve several aspects of fitness. Firefighting requires the men and women who choose this profession to possess a multitude of fitness aspects. Movements seen on the fireground tax the phosphagen and oxidative energy systems, and require muscular strength, muscular endurance, flexibility, and power. Given that firefighters are faced with an environment that is constantly varied and dynamic, HIFT may prove to be an effective training modality in this population. Implementing a physical fitness program that taxes the same energy systems and utilizes movements similar to firefighting tasks could not only improve fitness, but also improve movement literacy and quality which may lead to reductions in injuries.

## Figures and Tables

**Table 1:** Participant Descriptive and Fitness Statistics at Weeks 1.

Variable	Week 1
Sample (*N*)	89
Age (years)	26.8 ± 4.2
Body Mass (kg)	89.24 ± 16.33
Height (m)	1.78 ± 0.07
BMI (kg·m^−2^)	28.11 ± 4.19
Underweight (<18.5)	-
Normal (18.5–24.9)	22.5%
Overweight (25.0–29.9)	43.8%
Obese (>30)	33.7%
Cardiorespiratory Fitness ^†^ (ml∙kg^−1^∙min^−1^)	40.84 ± 5.09

Note. ^†^ Estimated VO_2max_ [32].

**Table 2:** Tentative Academy HIFT Schedule.

Week	Monday	Tuesday	Wednesday	Thursday	Friday
1	No PT	Academy Physical Fitness Test	Extended Warm-Up/Fitness Testing	Academy Firefighter Challenge	Team Challenge Battle Hose 1 ¾” Tire Triceps Dips Air Squats Team Push-ups
2	4 Stations Stair Run 2.5” Hose Drag Litter Carry Bear Crawl/Plank	Standard Body Weight Circuits	15-min Ability Group Run + Mobility/Flexibility	Partner Circuits Wall Ball/Plank Burpee/Wall-sit Tire Drag/Lunge with Press Box Step-up/Wall Ball Slams	Team Challenge Battle Hose 1 ¾” Tire Triceps Dips Air Squats Team Push-ups
3	Battle House Stations Alternate Arms/Air Squats Hip-to-hip/Push-ups Slam to Burpee/Glute Bridges Hose Jumping Jacks/Forearm to Wrist Planks	Standard Body Weight Circuits	20-min Ability Group Run + Mobility/Flexibility	Partner Circuits Wall Ball/Plank Sledge Strikes/Wall-sit Tire Drag/Lunge with Press Box Step-up/Wall Ball Slams	Team Challenge Alternate Dummy Drag 2 Lap Litter Carry w/Dummy 10 burpees per person
4	Academy Physical Fitness Test	4 Corners Army Crawl/Rest Bear Crawl/Inch Worm Crab Walk/Jump Rope Lunge w/Twist/Spiderman	20-min Ability Group Run + Mobility/Flexibility	4 Corners High Plank Hose Drag Hurdles/Rest Boat Pose w/Med Ball Rotation Hurdles/Rest	Memorial Stadium Stair Run
5	4 Stations Stair Run 2.5” Hose Drag Litter Carry Bear Crawl/Plank	Partner Circuits Battle Hose/Push-ups Farmer Carry w/2 ½” hose/Tire Triceps Dips Bear Crawl Hose Pull/Rest Box Step-ups/Bicycle Crunches	20-min Ability Group Run + Mobility/Flexibility	Partner Circuits Sledge Strikes/Wall-sit High Rise Bundle Carry/Crunches Front Squats/Forearm to Wrist Planks Burpees/Flutter Kick	Team Challenge Tire Drive/Pull Victim Drag 1 Lap Run Battle Hose Front Squats
6	Battle House Stations Alternate Arms/Air Squats Hip-to-hip/Push-ups Slam to Burpee/Glute Bridges Hose Jumping Jacks/Forearm to Wrist Planks	Partner Circuits Side Lunge with 1 ¾” Hose/Jump Rope Thrusters/Jumping Jacks Bear Crawl Hose Drag/Rest Box Step-ups/Bicycle Crunches	20-min Ability Group Run + Mobility/Flexibility	Memorial Stadium Challenge	Extended Warm-up/Fitness Testing
7	Academy Physical Fitness Test	Recovery/Motivation	Academy Firefighter Challenge	Evaluations	Graduation

**Table 3:** Previous Academy Daily Training Tentative Schedule.

Training Day	Exercise
Week 1	
Monday	No Physical Fitness Training
Tuesday	Academy’s Physical Fitness Test
Wednesday	Extended Warm up (Data Collection)
Thursday	Introduction to PT Program
Week 2	
Monday	Ability Group Run
Tuesday	Muscular Endurance Training
Wednesday	Ability Group Run
Thursday	Muscular Endurance Training
Week 3	
Monday	Stair Run/Dummy Drag
Tuesday	Muscular Endurance Training/Run
Wednesday	Ability Group Run
Thursday	Muscular Endurance Training
Week 4	
Monday	Stair Run/Dummy Drag
Tuesday	Circuit Training
Wednesday	Ability Group Run
Thursday	Muscular Endurance/Run
Week 5	
Monday	Gear March
Tuesday	Circuit Training
Wednesday	Ability Group Run
Thursday	Muscular Endurance
Week 6	
Monday	Academy Physical Fitness Test
Tuesday	Extended Warm-up
Wednesday	Data Collection
Thursday	Program Feedback

**Table 4:** Changes in Fitness and Firefighting Ability from Week-1 to Week-7.

Variable	Pre (M ± SD)	Post (M ± SD)	Mean Difference (Post-Pre)	% Change	*p*-Value	Effect Size (Cohen’s *d*)
Weight (kg)	89.2 ± 16.3	88.6 ± 15.2	−0.6	<1.0%	0.03	0.04
BMI	28.1 ± 4.2	27.9 ± 3.8	−0.2	<1.0%	0.04	0.05
1.5-mile (2.4 km) run (min·s)	13.1 ± 1.8	11.7 ± 1.5	−1.4	−10.7%	≤0.001	0.85
Estimated VO_2max_ (ml∙kg^−1^∙min^−1^)	40.8 ± 5.1	45.3 ± 5.2	4.5	11.0%	≤0.001	0.88
Push-ups (reps)	41.9 ± 12.4	57.5 ± 13.3	15.5	37.2%	≤0.001	1.22
Sit-ups (reps)	31.4 ± 6.1	38.3 ± 7.8	6.9	22.0%	≤0.001	0.99
YMCA bench press (reps)	30.4 ± 11.6	35.6 ± 11.6	5.2	17.1%	≤0.001	0.45
Flexibility (cm)	7.6 ± 7.2	9.8 ± 7.1	2.2	28.9%	≤0.001	0.31
Vertical Jump (in)	24.3 ± 3.7	24.4 ± 4.1	0.1	<1.0%	0.62	-
Keiser^®^ Sled (s)	44.3 ± 17.3	35.4 ± 10.7	−8.9	−20.1%	≤0.001	0.62
SCBA Crawl (s)	44.2 ± 11.7	35.2 ± 8.9	−9.0	−20.4%	≤0.001	0.87
Victim Drag (s)	22.5 ± 5.9	19.4 ± 4.6	−3.1	−13.8%	<0.001	0.59
Hose Advance (s)	15.2 ± 3.7	13.9 ± 3.7	−1.3	−8.6%	0.02	0.35
Equipment Carry	20.9 ± 3.2	19.3 ± 3.1	−1.6	−7.7%	≤0.001	0.51
Ladder Raise	7.4 ± 2.2	6.5 ± 1.5	−0.9	−12.2%	≤0.001	0.48
Challenge Total (s)	240.2 ± 41.2	192.5 ± 41.6	−47.7	−19.9%	≤0.001	1.16

Note: Cohen’s *d* effect size: 0.2 = small effect size; 0.5 = medium effect size; 0.8 = large effect size.

**Table 5:** Changes in Cardiorespiratory Fitness Classifications.

Estimated VO_2max_ Classification	Week 1	Week 7
Cardiorespiratory Fitness ^†^	40.84 ± 5.09	45.30 ± 5.24
Very poor *	11.2%	3.4%
Poor *	25.8%	6.7%
Fair *	51.7%	55.1%
Good *	10.1%	23.8%
Excellent *	1.1%	9.0%
Superior *	-	-

Note. ^†^ Estimated VO_2max_; * American College of Sports Medicine (ACSM) Cardiorespiratory Fitness Classifications [32].

**Table 6:** Changes in Fitness during HIFT and Traditional PT (PT).

Variable	HIFT % Δ	Effect Size (Cohen’s *d*)	PT % Δ	Effect Size (Cohen’s *d*)	*p*-Value
1.5-mile (2.4 km) run (min·s)	−10.70%	0.85	−7.9%	0.68	0.01
Estimated VO_2max_ (ml∙kg^−1^∙min^−1^)	11.0%	−0.88	7.8%	0.67	0.004
Push-ups (reps)	37.2%	−1.22	36.2%	1.17	0.14
Sit-ups (reps)	22.0%	−0.99	17.8%	0.62	0.023
YMCA bench press (reps)	17.1%	−0.45	9.3%	0.29	0.003
Flexibility (cm)	28.9%	−0.31	35.2%	0.36	0.27
ΔOverall Fitness	-	-	-	-	≤0.001

Note: The final column compares the post means for HIFT and TPT for the age and VO_2max_ matched controls.

## Data Availability

Data from this study is safely stored in the Exercise Psychophysiology Laboratory (ExPPL) at the University Illinois Urbana-Champaign.

## References

[1] National Fire Protection Association 1500 (2018). Fire Department Occupational Safety, Health, and Wellness Program.

[2] National Fire Protection Association 1583 (2015). Standard on Health-Related Fitness Programs for Fire Department Members.

[3] Storer T.W., Dolezal B.A., Abrazado M.L., Smith D.L., Batalin M.A., Tseng C.H., Cooper C.B. (2014). Firefighter health and fitness assessment: A call to action. J. Strength Cond. Res..

[4] Drew-Nord D.C., Myers J., Nord S.R., Oka R.K., Hong O., Froelicher E.S. (2011). Accuracy of peak VO2 assessments in career firefighters. J. Occup. Med. Toxicol..

[5] Gnacinski S.L., Ebersole K.T., Cornell D.J., Mims J., Zamzow A., Meyer B.B. (2016). Firefighters’ cardiovascular health and fitness: An observation of adaptations that occur during firefighter training academies. Work.

[6] Poplin G.S., Roe D.J., Burgess J.L., Peate W.F., Harris R.B. (2016). Fire fit: Assessing comprehensive fitness and injury risk in the fire service. Int. Arch. Occup. Environ. Health.

[7] Fogleman M., Bhojani F.A. (2005). Refinery Firefighters: Assessing Fitness for Duty. Int. J. Occup. Saf. Ergon..

[8] Jahnke S.A., Hyder M.L., Haddock C.K., Jitnarin N., Day R.S., Poston W.S.C. (2015). High-intensity Fitness Training Among a National Sample of Male Career Firefighters. Saf. Health Work.

[9] Phillips D.B., Scarlett M.P., Petersen S.R. (2017). The Influence of Body Mass on Physical Fitness Test Performance in Male Firefighter Applicants. J. Occup. Environ. Med..

[10] Roberts M.A., O’Dea J., Boyce A., Mannix E.T. (2002). Fitness levels of firefighter recruits before and after a supervised exercise training program. J. Strength Cond. Res..

[11] Smith D.L. (2011). Firefighter Fitness. Curr. Sports Med. Rep..

[12] Anderson A.A., Yoo H., Franke W.D. (2016). Associations of Physical Activity and Obesity with the Risk of Developing the Metabolic Syndrome in Law Enforcement Officers. J. Occup. Environ. Med..

[13] Kuehl K.S., Kisbu-Sakarya Y., Elliot D.L., Moe E.L., Defrancesco C.A., Mackinnon D.P., Lockhart G., Goldberg L., Kuehl H.E. (2012). Body mass index is a predictor of fire fighter injury and worker compensation claims. J. Occup. Environ. Med..

[14] Jitnarin N., Poston W.S., Haddock C.K., Jahnke S.A., Day R.S. (2014). Accuracy of Body Mass Index-defined Obesity Status in US Firefighters. Saf. Health Work.

[15] Patterson P.D., Smith K.J., Hostler D. (2016). Cost-effectiveness of workplace wellness to prevent cardiovascular events among U.S. firefighters. BMC Cardiovasc. Disord..

[16] Seyedmehdi S.M., Attarchi M., Cherati A.S., Hajsadeghi S., Tofighi R., Jamaati H. (2016). Relationship of aerobic fitness with cardiovascular risk factors in firefighters. Work.

[17] Griffin S.C., Regan T.L., Harber P., Lutz E.A., Hu C., Peate W.F., Burgess J.L. (2016). Evaluation of a fitness intervention for new firefighters: Injury reduction and economic benefits. Inj. Prev..

[18] Moore M.A. (2003). Cardiovascular disease: A continuing threat to homeland defense. J. Clin. Hypertens..

[19] Poston W.S., Jitnarin N., Haddock C.K., Jahnke S.A., Tuley B.C. (2011). Obesity and Injury-Related Absenteeism in a Population-Based Firefighter Cohort. Obesity.

[20] Msci D.P.P., Martínez-Vizcaíno V., López M.S., Bartolomé-Gutiérrez R., Rodríguez-Martín B., Notario-Pacheco B. (2017). Resilience as a mediator between cardiorespiratory fitness and mental health-related quality of life: A cross-sectional study. Nurs. Health Sci..

[21] Chizewski A., Box A., Kesler R., Petruzzello S.J. (2021). Fitness Fights Fires: Exploring the Relationship between Physical Fitness and Firefighter Ability. Int. J. Environ. Res. Public Health.

[22] Yang J., Teehan D., Farioli A., Baur D.M., Smith D., Kales S.N. (2013). Sudden Cardiac Death Among Firefighters ≤45 Years of Age in the United States. Am. J. Cardiol..

[23] Kesler R.M., Ensari I., Bollaert R.E., Motl R.W., Hsiao-Wecksler E., Rosengren K.S., Fernhall B., Smith D.L., Horn G.P. (2017). Physiological response to firefighting activities of various work cycles using extended duration and prototype SCBA. Ergonomics.

[24] Fyock-Martin M.B., Erickson E.K., Hautz A.H., Sell K.M., Turnbaugh B.L., Caswell S.V., Martin J.R. (2020). What do Firefighting Ability Tests Tell Us about Firefighter Physical Fitness? A Systematic Review of the Current Evidence. J. Strength Cond. Res..

[25] Pawlak R., Clasey J.L., Palmer T., Symons T.B., Abel M.G. (2015). The Effect of a Novel Tactical Training Program on Physical Fitness and Occupational Performance in Firefighters. J. Strength Cond. Res..

[26] Crawford D.A., Drake N.B., Carper M.J., DeBlauw J., Heinrich K.M. (2018). Are Changes in Physical Work Capacity Induced by High-Intensity Functional Training Related to Changes in Associated Physiologic Measures?. Sports.

[27] Feito Y., Heinrich K.M., Butcher S.J., Poston W.S.C. (2018). High-Intensity Functional Training (HIFT): Definition and Research Implications for Improved Fitness. Sports.

[28] Haddock C.K., Poston W.S.C., Heinrich K.M., Jahnke S.A., Jitnarin N. (2016). The Benefits of High-Intensity Functional Training Fitness Programs for Military Personnel. Mil. Med..

[29] Heinrich K.M., Spencer V., Fehl N., Poston W.S.C. (2012). Mission Essential Fitness: Comparison of Functional Circuit Training to Traditional Army Physical Training for Active Duty Military. Mil. Med..

[30] Abel M.G., Mortara A.J., Pettitt R.W. (2011). Evaluation of Circuit-Training Intensity for Firefighters. J. Strength Cond. Res..

[31] Abel M.G., Palmer T.G., Trubee N. (2015). Exercise Program Design for Structural Firefighters. Strength Cond. J..

[32] Dumke C.L., Riebe D. (2008). Health-Related Physical Fitness Testing and Interpretation. ACSM’s Guidelines for Exercise Testing and Prescription.

[33] Golding L.A., Myers C.R., Sinning W.E. (1989). Y’s Way to Physical Fitness.

[34] National Strength and Conditioning Association (2012). NSCA’s Guide to Tests and Assessments.

[35] Maxell S. (2001). Analysis of variance. J. Consum. Psychol..

[36] Staley J.A., Weiner B., Linnan L. (2011). Firefighter fitness, coronary heart disease, and sudden cardiac death risk. Am. J. Health Behav..

[37] Cornell D.J., Gnacinski S.L., Meyer B.B., Ebersole K.T. (2017). Changes in health and fitness in firefighter recruits: An observational cohort study. Med. Sci. Sports Exerc..

[38] Peterson M.D., Dodd D.J., Alvar B.A., Rhea M.R., Favre M. (2008). Undulation Training for Development of Hierarchical Fitness and Improved Firefighter Job Performance. J. Strength Cond. Res..

[39] Del Sal M., Barbieri E., Garbati P., Sisti D., Rocchi MB L., Stocchi V. (2009). Physiologic responses of firefighter re-cruits during a supervised live-fire work performance test. J. Strength Cond. Res..

[40] Soteriades E.S., Smith D.L., Tsismenakis A.J., Baur D.M., Kales S.N. (2011). Cardiovascular disease in US firefighters: A systematic review. Cardiol. Rev..

[41] Kim P.S., Mayhew J.L., Peterson D.F. (2002). A modified YMCA bench press test as a predictor or 1 repetition maximum bench press strength. J. Strength Cond. Res..

